# Massachusetts Dental Society

**Published:** 1904-01

**Authors:** Waldo E. Boardman

**Affiliations:** Editor


### MASSACHUSETTS DENTAL SOCIETY.

(Continued from Vol. XXIV., page 893.)

*Thursday, June 4, 1903.*

The President introduced Dr. Ned A. Stanley, D.M.D., of New Bedford, who read a paper entitled "The Advantages of Co-operation in our Profession."

(For Dr. Stanley's paper, see page 43.)

### DISCUSSION.

*Luther D. Shepard, A.M., D.D.S., D.M.D.,* Boston.—I had the pleasure of reading this paper at my leisure, and I am so well satisfied with it that all I could say would be a word of approbation. It covers the ground, it is concisely expressed, and it states the truth as I have seen it throughout my whole life.

If one would look over the literature of the profession for the past fifty years, he will be convinced that co-operation, the meeting together in societies, has been a great factor for individual growth of the man, extension of his reputation, and the enlargement of the profession by the interchange of valuable ideas.

I could give the names of hundreds who have been active dental society men, and I recall a few instances where men have made their reputation or have impressed themselves upon the world of progress through their activity as society members.

This is such a truism that I wonder at the professional men who keep apart from these meetings. There are among the living a multitude who could be named, but I prefer to speak only of the dead. Our late departed friend, Dr. McKellops, of St. Louis, probably during forty or fifty years gave at least one-twelfth of his time and labor to professional society work. He knew more men and more men knew him throughout the country, and his influence was greater for progress, than any one with whom I have been associated. I could give other examples, but it is not necessary.

The rubbing of man against man, thus reducing their sharp angles, comes from association and co-operation, and this, it seems to me, is a very important factor in the production of substantial growth.

The President introduced Frank R. Mayer, D.D.S., of Worcester, Mass., who read a paper on "Pyorrhœa Alveolaris."

(Dr. Mayer's paper will be published in February.)

The President called upon Dr. Walter E. Decker, D.D.S., of Boston, Mass., who read a paper entitled "A Card System for keeping Dental Records and Accounts."

(For Dr. Decker's paper, see page 19.)

The President then introduced Henry J. Janlusz, D.D.S., of New York City, who read a paper entitled "Suppurative Cleft Palates and Devices for the Same."

(Dr. Janlusz's paper will be published in February.)

There being no discussion on the above papers, the Association then adjouned *sine die.*

WALDO E. BOARDMAN, D.M.D.,
*Editor.*

